# Geographic Disparities in Declining Rates of Heart Disease Mortality in the Southern United States, 1973–2010

**DOI:** 10.5888/pcd11.140203

**Published:** 2014-10-23

**Authors:** Adam S. Vaughan, Michael R. Kramer, Michele Casper

**Affiliations:** Author Affiliations: Michael R. Kramer, Rollins School of Public Health, Emory University, and Centers for Disease Control and Prevention, Atlanta, Georgia; Michele Casper, Centers for Disease Control and Prevention, Atlanta, Georgia.

**Figure:**
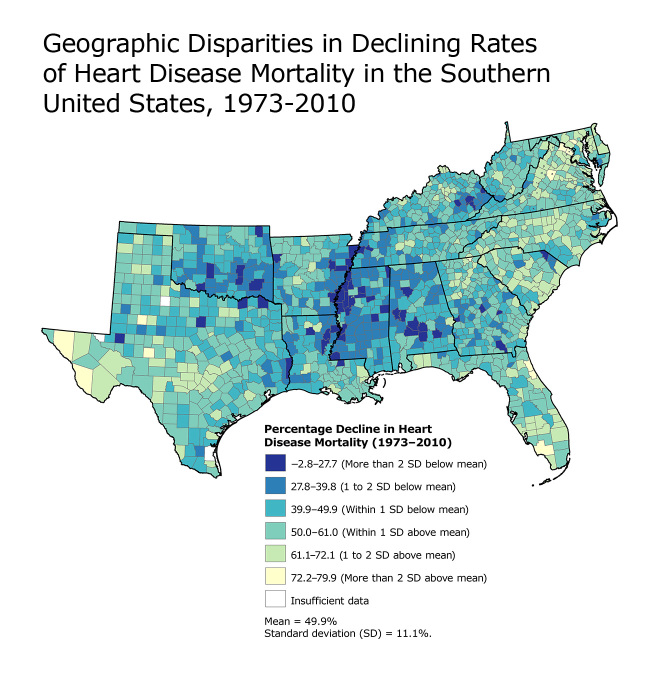
This map shows model-based, county-level percentage decline in heart disease death rates from 1973 to 2010 in the Southern United States. During this 37-year period, the fastest declines (in yellow) occurred primarily on the East Coast and central and west Texas, and the slowest declines (in dark blue) were concentrated largely in the counties along the Mississippi River and parts of Kentucky, Oklahoma, and Alabama, which are also areas characterized by extremely high spatially concentrated poverty rates (6). Data source: National Center for Health Statistics.

## Background

In the United States, heart disease mortality has decreased since the 1950s (1). However, these declines have not occurred uniformly across the country, and slower rates of decline may indicate increased need for heart disease prevention policies and programs (2). In this article, we document the geographic inequalities in declining heart disease death rates in the South — a region with high heart disease mortality rates. We also consider methods for generating county-level estimates of changing heart disease death rates.

## Methods

Our analysis was limited to 1,420 counties in the South US Census Region. The annual number of county-level heart disease deaths from 1973 to 2010 among people aged 35 years or older was obtained from the National Center for Health Statistics (NCHS). Heart disease deaths were defined according to the following International Classification of Diseases (ICD) codes: ICD-8: 390–398, 402, 404, 410–429; ICD-9: 390–398, 402, 404–429; ICD-10: I00–I09, I11, I13, I20–I51. County-level population estimates were also obtained from the NCHS Centers for Disease Control and Prevention (CDC) WONDER database. These data were sequentially 1) aggregated into 2-year intervals, 2) age-standardized (using the 2000 US standard population), and 3) spatially smoothed using Bayesian methods, with the first and third steps enhancing rate stability of counties with small populations (3). Only counties with 2-year populations greater than 1,000 at all intervals were included.

These stabilized, age-standardized, biennial county-level heart disease death rates were modeled using a spatially independent Poisson generalized linear mixed model with a fixed intercept and temporal slope and county-level random intercept and temporal slope using SAS version 9.3 (SAS Institute, Inc) (4). The assumed log-linear trend was verified using joinpoint regression (Joinpoint Regression Program version 4.0.4, National Cancer Institute). Given this model, county-level temporal changes (ie, percentage declines) in heart disease death rates were summarized as the exponentiated linear combination of the fixed and random slopes. The resulting county-level percentage declines in heart disease death rates (1973–2010) were mapped (ArcMap, ESRI), with categories defined according to the mean and standard deviation.

## Main Findings

This map illustrates substantial geographic disparities in declining heart disease death rates in the South from 1973 to 2010. Although the mean county-level percentage decline in heart disease mortality was 49.9% (standard deviation, 11.1%), the magnitude of change varied among counties from a decline of 79.9% to an increase of 2.8%. The largest declines were concentrated in central and west Texas and the Atlantic Coast, and the smallest declines were observed in counties along the Mississippi River, eastern Kentucky, and parts of Oklahoma and Alabama.

## Action

This map illustrates impressive reductions in heart disease mortality in most counties in the South during the past 40 years. By employing statistical models widely used in public health, we have summarized multiple years of data into a single, readily interpretable value, enabling greater understanding of the geographic variation in declining heart disease mortality. The wide range and geographic patterning of declining heart disease death rates in the South reinforces the importance of place on the population burden of heart disease (5). Areas with slower rates of decline, including parts of the Mississippi Delta, Kentucky, Oklahoma, and Alabama, suggest the need for enhanced heart disease prevention efforts. Future research is needed to better understand the observed spatial variation in declining heart disease mortality, including exploration of disparities in declines by race, sex, and social context.
